# Optimization of Se- and Zn-Enriched Mycelium of *Lentinula edodes* (Berk.) Pegler as a Dietary Supplement with Immunostimulatory Activity

**DOI:** 10.3390/nu15184015

**Published:** 2023-09-16

**Authors:** Małgorzata Kałucka, Aleksander Roszczyk, Marzenna Klimaszewska, Beata Kaleta, Ewelina Drelich, Anna Błażewicz, Sandra Górska-Jakubowska, Eliza Malinowska, Marek Król, Aleksandra Maria Prus, Katarzyna Trześniowska, Aleksandra Wołczyńska, Przemysław Dorożyński, Radosław Zagożdżon, Jadwiga Turło

**Affiliations:** 1 Department of Drug Technology and Pharmaceutical Biotechnology, Medical University of Warsaw, Banacha 1, 02-097 Warsaw, Poland; malgorzata.kalucka@wum.edu.pl (M.K.); przemyslaw.dorozynski@wum.edu.pl (P.D.); 2 Department of Clinical Immunology, Medical University of Warsaw, Nowogrodzka 59, 02-006 Warsaw, Poland; aleksander.roszczyk@wum.edu.pl (A.R.); beata.kaleta@wum.edu.pl (B.K.);; 3 Faculty of Pharmacy, Medical University of Warsaw, 02-097 Warsaw, Poland; drelichewelina@gmail.com (E.D.);; 4 Department of Pathobiochemistry and Interdisiciplinary Applications of Ion Chromatography, Medical University of Lublin, Chodźki 1, 20-093 Lublin, Poland; annablazewicz@umlub.pl; 5 Department of Spectrometric Methods, National Medicines Institute, Chełmska 30/34, 00-725 Warsaw, Poland; k.trzesniowska@nil.gov.pl (K.T.); a.wolczynska@nil.gov.pl (A.W.); 6 Department of Immunology, Transplantology and Internal Medicine, Medical University of Warsaw, Nowogrodzka 59, 02-006 Warsaw, Poland

**Keywords:** zinc–selenite interactions, polysaccharides, *L. edodes*, immunomodulation

## Abstract

Mycelial cultures of *Lentinula edodes*, an edible and medicinal mushroom, have been used in our previous research to obtain selenium-containing immunomodulatory preparations. Our current attempts to obtain a new preparation containing both selenium and zinc, two micronutrients necessary for the functioning of the immune system, extended our interest in the simultaneous accumulation of these elements by mycelia growing in media enriched with selenite and zinc(II) ions. Subsequently, we have studied the effects of new *L. edodes* mycelium water extracts with different concentrations of selenium and zinc on the activation of T cell fraction in human peripheral blood mononuclear cells (PBMCs). Flow cytometry analysis was used to measure the expression of activation markers on human CD4+ and CD8+ T cells stimulated by anti-CD3 and anti-CD3/CD28 antibodies (Abs). It was demonstrated that statistically significant changes were observed for PD-1 and CD25 antigens on CD8+ T cells. The selenium and zinc content in the examined preparations modified the immunomodulatory activity of mycelial polysaccharides; however, the mechanisms of action of various active ingredients in the mycelial extracts seem to be different.

## 1. Introduction

The global coronavirus pandemic has put pressure on researchers to develop, test, and deploy active agents for use in the treatment of viral infections. Apart from medicines that act directly on the viruses, immune-stimulating preparations also seem to be interesting in the context of treating and preventing viral infections. Numerous reports on the immunomodulatory activity of preparations containing zinc and selenium show that supplementation with these micronutrients may be beneficial for both the prevention and treatment of viral infections, including coronaviruses [1]. Dietary multi-micronutrient supplements containing selenium dosed at up to 200 µg/day have potential as safe, inexpensive, and widely available adjuvant therapy in viral infections, e.g., human immunodeficiency virus (HIV) and influenza A virus (IAV). They can also be used in case of coinfections with HIV and Mycobacterium tuberculosis to support chemotherapy and to improve patients’ quality of life [2,3,4,5,6].

Zinc status, like selenium, is a critical factor that can influence antiviral immunity; zinc-deficient populations are often most at risk of acquiring viral infections such as HIV or hepatitis C virus [7,8].

The biological availability of zinc and selenium from pharmaceutical preparations are limited by many factors, including both those that are intrinsic and extrinsic to the host. For both micronutrients, bioavailability, tissue distribution, and toxicity strongly depend on the form ingested. In general, organic forms have higher bioavailability and/or lower toxicity than inorganic species [9,10,11]. While both zinc and selenium play a well-documented role in cancer prevention (e.g., for prostate cancer) and adjuvant therapy in viral infections (e.g., HIV, IAV), their combined supplementation is often given as a recommended prophylactic agent [12,13].

An interesting hypothesis that has been continuously verified by researchers over several years is the synergism between the immunomodulatory effect of selenium (probably also zinc) and polysaccharides—mostly β-glucans of natural origin. Several members of the Basidiomycota division, often referred to as “medicinal mushrooms”, can produce immunomodulating substances, which are usually polysaccharides [14,15,16,17]. Fungal polysaccharides are used as a non-invasive form of cancer treatment due to their ability to induce a non-specific response of the immune system against cancer cells. Further, this capacity is also evident against viral and bacterial infections and inflammation [18]. *Lentinula edodes* is among the most well-studied fungi capable of biosynthesis of the most active immunostimulatory polysaccharides (e.g., lentinan) and is used in several countries in the treatment of cancer, HIV, hepatitis, and other diseases related to immunodeficiency. Further, fungi are unique in the accumulation of trace elements, which is a focus of interest of several research groups [19,20,21,22,23,24]. Thus, the submerged cultures of *Lentinula edodes* have been used in our previous research to obtain new immunomodulatory preparations enriched in selenium. We assumed that this would produce a synergistic effect between the polysaccharide compounds and selenium, which has been confirmed by the already published studies [25,26,27].

In continuation, we have designed a new preparation containing immune-active polysaccharides and both selenium and zinc, two micronutrients necessary for the functioning of the immune system. The necessity to design a biotechnological process for cultivating *L. edodes* mycelial cultures enriched in Zn and Se has extended our interest in the interactions between selenite and zinc (II) ions in the substrate and their effects on the transport of these ions into the fungal cell. In fungi, the process of the accumulation of ions from solutions consists of three phases: biosorption on the surface of the fungal cell wall, intracellular uptake, and chemical transformation [28,29]. For the examination of the last two processes, living fungal biomass is required. Culture media contaminated with heavy metals usually have a very complex composition, with the result that metals’ biosorption may be affected by the presence of other ions. Moreover, the mechanisms of intracellular uptake and chemical transformation differ for cations and anions. For the zinc(II) ions and selenites investigated in this work, the mechanisms of accumulation by the fungus are also diverse and, in higher mushrooms, remain poorly understood. An examination of the mechanisms of zinc(II) transport in Saccharomyces cerevisiae revealed the central role of the ZIP (Zrt- or Irt-like protein) family of zinc transporters [30,31]. Selenites in yeast were found to be absorbed in a metabolism-dependent way, using a transporter of phosphate or monocarboxylate [32,33,34]. Gharieb and Gadd [32] also reported a fast, metabolism-independent process during selenite uptake in *S. cerevisiae*. Carrier-mediated passive transport might be crucial to selenite absorption by *Flammulina velutipes* [35].

In our previous studies, we found that the introduction of selenites into the medium strongly affected heavy metal uptake in mycelia. Interestingly, the effect was variable for different ions. For Ni(II) and Fe(III) ions, the metal content in harvested mycelia rose in proportion to the selenite concentration in the culture medium. However, the Zn content in harvested mycelia decreased when the concentration of selenites in the medium rose [24,36]. Importantly, in our experiments, the molarities of Zn(II) and SeO_3_^2−^ in medium were not high enough to precipitate ZnSeO_3_ into the medium [37]. Most likely, the inhibition of zinc accumulation by selenites (and vice versa) resulted from the formation of soluble Zn(II)–selenite complexes in the culture medium. The phenomenon of the formation of Zn(II)–selenite complex compounds with different solubility, strength, and charge, depending on the molar ratio of ions, has been described by Banks, Moriya and Sekine, Somer and Yilmaz, and Feroci et al. [38,39,40,41]. Thus, determining the molar proportion of selenites and zinc (II) for the transport to the cell of both micronutrients has become an important task.

Considering all the factors described above, to obtain the new immunomodulatory preparation containing mushroom-derived polysaccharides, selenium, and zinc, we designed a multi-step approach:

- In the first step, we optimized the composition of the culture medium to obtain a *L. edodes* mycelia enriched in Se and Zn at the assumed concentrations;
- Then, we prepared mycelial extracts with defined polysaccharide, selenium, and zinc contents to test the expected biological activity;
- In the last stage of the work, we determined the effects of *L. edodes* mycelium extracts with different concentrations of selenium and zinc on human T cells activation.

## 2. Materials and Methods

### 2.1. Microorganism and Cultivation Media

The *Lentinula edodes* (Berk.) Pegler strain used in this study was ATCC 48085. The seed culture was grown under conditions described in our previous works [7].

#### Supplementation of Media and Growth Conditions in Mycelial Shake-Flask Cultures

The culture media contained glucose at 5%, yeast extract at 1%, soybean extract at 1.5%, and KH_2_PO_4_ at 0.1% (*w*/*v*). The pH of the medium was 6.5. Mineral precursors (sodium selenite and/or zinc bromide) were added to the medium, as shown in Table 1.

As a reference, we also performed a series of studies in which the cultivation medium was not enriched. The zinc and selenium contents in the unenriched medium were the control levels in these studies.

Mycelia were grown in shake-flask cultures in 500 mL flasks containing 200 mL of medium. The fermentation medium was inoculated with 5% (*v*/*v*) of the seed culture. Cultures were incubated at 26 °C in a rotary shaker (New Brunswick Scientific, Edison, NY, USA) at 120 rev/min for 10 days. Mycelia were harvested via filtration, washed three times with redistilled water, and freeze-dried. All experiments were conducted five times to ensure reproducibility.

### 2.2. Determination of Selenium and Zinc in L. edodes Mycelium

The determinations were performed after microwave mineralization.

#### 2.2.1. Microwave Mineralization Procedure

A microwave digestion procedure was performed using a closed-vessel microwave system (Magnum II, ERTEC). A total of 0.02 g of sample was placed in a Teflon container and digested with 3 mL of 65% HNO_3_ (Suprapur quality, Merck, Darmstadt, Germany) in the microwave digestion system according to a four-stage program: 17–20 atm for 3 min (60% of the microwave power), 27–30 atm for 5 min (80% of the microwave power), 42–45 atm for 8 min (100% of the microwave power), and cooling for 10 min. When cool, the sample was diluted to 25 mL with deionized water. If necessary, the solutions were diluted several times. A blank digest was prepared in parallel.

#### 2.2.2. Determination of Zinc Content by High-Performance Ion Chromatography (HPIC)

Chromatographic analyses were performed as described previously [24] on a Dionex DX-500 ion chromatograph (Dionex, Sunnyvale, CA, USA) equipped with an IP 25 isocratic pump, an IonPac CG 5A guard column, an IonPac CS 5A analytical column (250 × 4.6 mm I.D., 9 µm bead diameter, ethylvinylbenzene functionalized with both quaternary ammonium and sulfonate functional groups), a 25 μL injection loop, and a Dionex AD20 absorbance detector with a post-column pneumatic controller (PC 10) reactor. All samples were injected at least in triplicate. All measurements were made at 30 ± 1 °C. The eluent solution was 80 mM oxalic acid/100 mM tetramethylammonium hydroxide/50 mM potassium hydroxide at pH 4.7. The eluent flow rate was set to 0.3 mL/min. A solution of 4-(2-pyrridylazo)resorcinol (PAR) was used as the post-column reagent and its flow rate was set to 0.15 mL/min. The Dionex PeakNet chromatography workstation was used for instrument control and data acquisition.

#### 2.2.3. Determination of the Selenium Content via Reverse-Phase High-Performance Liquid Chromatography (RPHPLC)

The reverse-phase high-performance liquid chromatography method used for the determination of selenium was performed as described previously [41]. Namely, a 0.1% solution of diaminonaphthalene (DAN) in 0.1 M HCl was prepared and stored in the dark at 4 °C. The pH of each of digested sample dissolved in 8 mL of Suprapur water was adjusted to 1.8–2 by adding HCl or 7 M NH_3_·H_2_O, as needed. One milliliter of DAN was added to each sample, and the mixtures were heated for 45 min at 75 °C. After cooling, 3 mL of cyclohexane was added, and the samples were shaken vigorously for 1 min to extract the fluorescent piazselenol. Prior to RP HPLC determination, the samples were stored in the dark. The sample solutions were diluted with cyclohexane as needed. A blank digest was manipulated similarly for use as a control. Selenium standard solutions (6.25–1000 ng Se/mL) were prepared under the same conditions. The fluorescence was a linear function of the concentration of Se in the tested range (correlation coefficient, R = 0.999). The RP HPLC conditions were as follows: eluent, acetonitrile: flow rate, 1.4 mL/min; temperature, 25 °C; and injection volume, 20 μL. For fluorometric analysis, the excitation wavelength was 378 nm and the emission wavelength was 557 nm. The piazselenol retention time was 3.1 min.

### 2.3. Determination of the Selenium and Zinc Content in Mycelial Water Extracts via Inductively Coupled Plasma Mass Spectrometry (ICP-MS)

The solvents and reagents used for the test had the highest purity class available on the market. Ultrapure water (18 MΩ cm^−1^ resistivity) was taken from the Barnstead NANOPURE DIAMON UV System and used to prepare all standards and sample solutions. Suprapur 65% HNO_3_ grade was used to dissolve the samples. A 10 µg/mL concentration multi-element solution was purchased from INORGANIC Ventures (Christiansburg, VI, USA). The purity of the plasma gas (argon) and cell gas (helium) was greater than 99.999%.

Each sample was placed directly in the vessels sealed in the microdevice. Then, 3 mL of HNO_3_ was added. The mineralization of the samples was carried out in a high-pressure laboratory microwave oven (Milestone UltraWAVE T640). The heating program was carried out in two steps: In the first step, the temperature was increased linearly from 25 to 210 °C over 15 min. In the second stage, the temperature was kept at 210 °C for 8 min. After mineralization, the samples were diluted with water to a final volume of 100 mL. The Zn (Se) sample was further diluted (from 5 mL to 50 mL)

The quadrupole ICP-MS 7800 (Agilent Technologies, Tokyo, Japan) equipped with an eight-field collision chamber was used for element analysis. The internal standard (In) was added to compensate for any effects of acids or instrument drift. Measurements were made with a nickel probe and skimmer cones. The ICP-MS operating conditions are summarized in Table 2.

### 2.4. Preparation of the Mycelial Extracts

Hot water extracts were prepared from freeze-dried mycelium in a Soxhlet extractor. In order to investigate the effect of the micronutrient concentration ratio (zinc and selenium) on the immunomodulatory activity of the preparations, the following mycelial samples were selected for extraction:

- Mycelium cultured in medium not enriched with micronutrients (sample 0);
- Mycelium cultured in medium enriched with selenium only at a concentration of 0.8 mM (sample Se);
- Mycelium cultured in medium enriched with zinc only at a concentration of 0.8 mM (sample Zn);
- Mycelium cultured in medium enriched with selenium at a concentration of 0.8 mM and zinc at a concentration of 0.2 mM (sample Se/Zn);
- Mycelium enriched with selenium at a concentration of 0.2 mM and zinc at a concentration of 0.8 mM (sample Zn/Se).

After 8 h, during which the extraction of 0.5 g of mycelium with 20 mL of water occurred, the extract was evaporated under the reduced pressure. The freeze-dried extracts were used to test for chemical composition (polysaccharide, Zn, and Se contents) and redissolved in water to the appropriate concentration to test for biological activity.

### 2.5. Determination of the Carbohydrate Content in the Mycelial Extracts

The determination was performed using the phenol–sulphuric acid method (Dubois method) [42].

One milliliter of the sample (water extract at a concentration of 0.3 mg/mL) was mixed with l mL of 5% phenol solution and 5 mL of concentrated H_2_SO_4_. The mixture was kept at room temperature for 10 min and then heated at 30 °C for 20 min. Absorbance at 490 nm was measured. A calibration curve was constructed with different concentrations of 1:1 w/w galactose–mannose mixture (25–100 µg/mL) as the standard.

### 2.6. Determination of the Effects of Mycelial Extracts on T Cell Activation

#### 2.6.1. The Isolation, Culture, and Stimulation of PBMCs

Blood samples (8 mL) were collected from seven healthy donors. All blood samples were commercially obtained from the Regional Blood Centre in Warsaw. The isolation of peripheral blood mononuclear cells (PBMCs) was performed via density gradient centrifugation on a Histopaque-1077 (Sigma-Aldrich, St. Louis, MO, USA). PBMCs were collected at the interface between the plasma and the Histopaque and washed twice with phosphate buffered saline (PBS, Sigma-Aldrich, St. Louis, MO, USA). The separated PBMCs were resuspended in RPMI 1640 medium (Gibco, Gaithersburg, MD, USA) containing antibiotic-antimycotic solution (1.5% penicillin-streptomycin-amphotericin, Invitrogen, Waltham, MA, USA) and 10% human serum (Gibco, Gaithersburg, MD, USA) and counted. The PBMCs (2 × 105 cells/well) were cultured in 96-well flat-bottom microplates (Nunc, Paris, France), as described in detail in our previous paper [43]. Briefly, the cells were stimulated with anti-CD3 Ab (coated on plate wells, 0.75 μg/mL, BD Pharmingen, Franklin Lakes, NJ, USA) or Dynabeads™ Human T-Activator CD3/CD28 (2 μL per well, ratio 2:5, Gibco, Gaithersburg, MA, USA) and incubated with *L. edodes* mycelium extracts at a concentration of 100 µg/mL. The PBMCs were cultured for 24 h at 37 °C and 5% CO_2_ in a humidified incubator.

#### 2.6.2. Flow Cytometry

For flow cytometry, 2 × 105 cells from each well were washed once in PBS and stained with antibodies against specific surface antigens (all from BD Biosciences, San Jose, CA, USA): CD3-PerCP (SK7 clone), CD4-APC-Cy7 (SK3 clone), CD8-APC (SK1 clone), CD69- BV711 (FN50 clone, Horizon™), and CD25- FITC (2A3 clone). Prior to the experiments, each antibody was titrated in order to achieve the highest signal-to-background ratio. Samples were incubated in the dark for 15 min at room temperature. Next, cells were rinsed in 1 mL of PBS without Ca^2+^ or Mg^2+^ and with 0.01% sodium azide and resuspended in 60 μL PBS without Ca^2+^ or Mg^2+^ and with 0.01% sodium azide. Cut-off values for each positive population were based on fluorescence minus one experiment.

Relative frequencies of CD69 and CD25 expression were assessed on singlet populations of CD3+CD4+ T cells and CD3+CD8+ T cells. Flow cytometry was performed using a DxFLEX Flow Cytometer (Beckman Coulter, Brea, CA, USA) and analyzed using CytExpert software.

### 2.7. Statistical Analysis

Statistical analyses (biotechnological and analytical part) were performed using the StatSoft, Inc. (Tulsa, OK, USA) (2014) STATISTICA (data analysis software system), version 12. All computations were applied at a significance level of 0.05.

All data in groups were first tested for normality using the Shapiro–Wilk test. To compare the means of two groups, a t-test was applied if the variances of the two populations were equal; otherwise, a Cochran–Cox test was applied. To check the homogeneity of variance, the Brown–Forsythe test was used.

Statistically significant differences between more than two groups were assessed using one-way analysis of variance, which was preceded by checking the assumptions of normality and homogeneity of variance. For multiple comparisons, the Tukey post-hoc test was performed; meanwhile, to compare the control group to each of the others, Dunnett’s test was used.

Flow cytometry data were acquired and interpreted using CytExpert version 2.4 software (Beckman Coulter, Miami, FL, USA). The acquired data were then analyzed and visualized with GraphPad Prism 9.5.0 software. To determine the normality of the data sets, Shapiro–Wilk and Kolmogorov–Smirnov tests were applied using α = 0.05, and each set was considered to have normal distribution when both tests showed *p* > 0.05. To compare the data sets, repeated measures ANOVA tests with Geisser–Greenhouse correction were applied to data sets with normal distribution, while Friedman tests were conducted for those without normal distributions, both using α = 0.05. Results were considered significantly important when *p* < 0.05.

## 3. Results

### 3.1. Accumulation of Se and Zn in the Mycelium of L. edodes

#### 3.1.1. Reference Series and Mycelial Growth

The cultivation of the mushroom mycelium in medium not enriched in Na_2_SeO_3_ and/or ZnBr_2_ afforded 7–8 g of dry-weight mycelium per liter of culture medium after 10 days. The selenium and zinc concentrations of the medium not enriched in sodium selenite and zinc bromide medium were 1.138 µM and 0.036 mM, respectively. The determination was performed after mineralization of a medium sample with concentrated nitric acid. The total Se and Zn contents were determined without speciation.

The selenium and zinc contents of the reference series of dried mycelium (mycelium cultivated in unenriched medium) equaled 1.27 and 289 µg/g, respectively. The mycelial growth in enriched media was only weakly affected by increasing the concentrations of the supplements.

**Figure 1:**
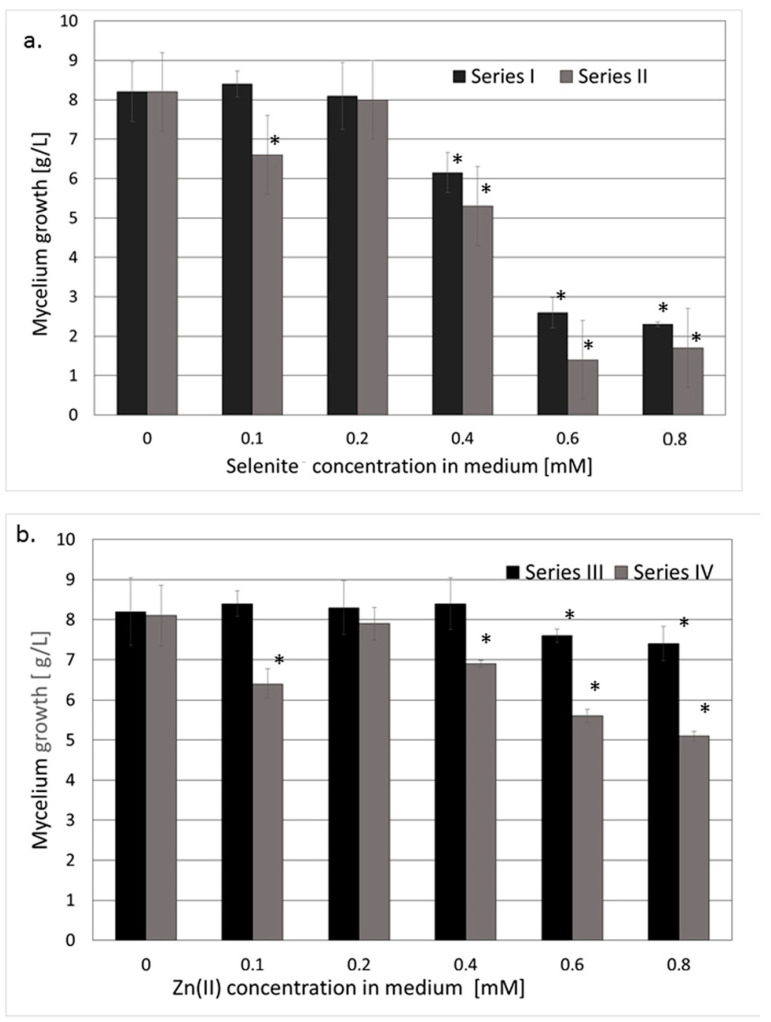
The mycelial growth of *L. edodes* (**a**) in a medium enriched with selenites exclusively (Series I) versus in a medium also containing Zn(II) (Series II); (**b**) in a medium enriched with Zn(II) exclusively (Series III) versus in a medium also containing selenites (Series IV). (*) values significantly different from the reference culture cultivated in non-enriched medium (*p* < 0.05).

A depressive effect was observed only in cultures enriched with sodium selenite or zinc bromide in concentrations higher than 0.4 mM. The mycelium growth significantly decreased when the second ion (selenite or zinc) was added to the medium (Figure 1a,b), except for the cultures with a zinc(II)/selenite molar ratio of 1:1.

#### 3.1.2. The Accumulation of Se Exclusively versus in the Presence of Zn(II)

**Figure 2:**
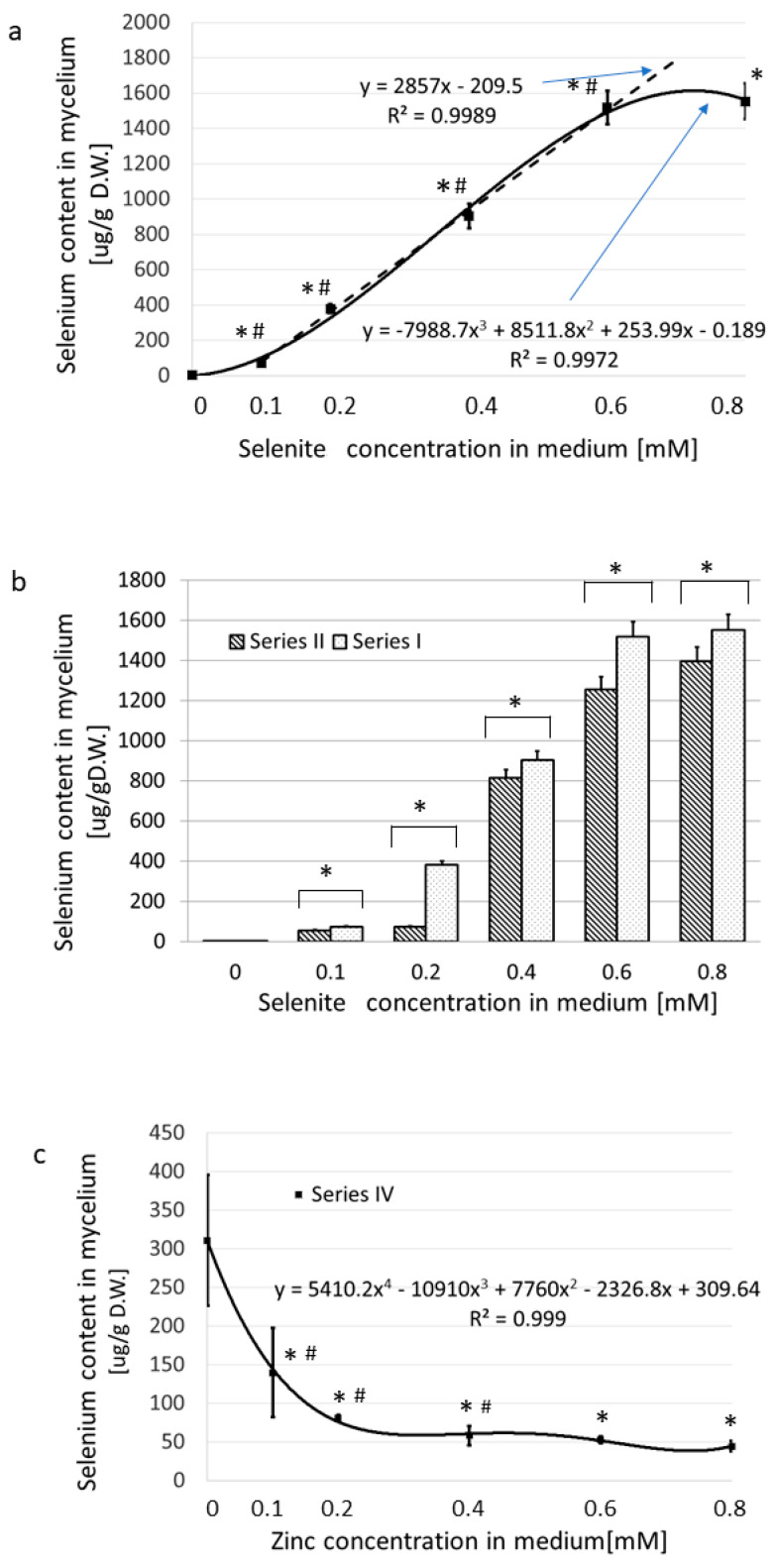
Accumulation of selenium into the mycelium of *L. edodes* from a medium enriched in selenites exclusively versus in a medium also containing Zn(II). (**a**) The content of selenium absorbed by the mycelium as a function of the selenite concentration in a medium enriched exclusively with sodium selenite. (*) values significantly different to the reference culture cultivated in non-enriched medium (*p* < 0.05). (#) values significantly different to the previous culture supplemented with a lower concentration of selenite (*p* < 0.05). (**b**) Comparison of Se accumulation from medium enriched exclusively with selenites (Series I) versus in the presence of 0.2 mM Zn(II) (Series II). (*) values of Series I significantly different to Series II (*p* < 0.05). (**c**) Impact of increasing Zn(II) concentration in the medium (0–0.8 mM) on the accumulation of selenium from a medium enriched in 0.2 mM sodium selenite (Series IV). (*) values significantly different to the reference culture cultivated in non-enriched medium (*p* < 0.05). (#) values significantly different to the previous culture supplemented with a lower concentration of selenite (*p* < 0.05). In each panel, the data are presented as the mean ± S.D. of five observations.

In the Series I (medium supplemented with Na_2_SeO_3_), the uptake of selenium to the submerged cultivated mycelium was very effective, as was also found in our previous works [44,45]. The addition of 0.8 mmol/L of sodium selenite to the medium (the maximum concentration used) yielded 1554 µg of selenium per 1 g of dry-weight mycelium (Figure 2a, Table S1).

The content of selenium in the dry-weight mycelium correlated well with the Na_2_SeO_3_ concentration in the medium for concentrations ranging from 0.1 to 0.6 mM. At levels higher than 0.6 mM, no additional uptake of Se from the medium occurred (Figure 2a).

In Series II (medium enriched in Na_2_SeO_3_ and containing zinc bromide at 0.2 mM), equimolar concentrations of zinc and selenites in the medium resulted in an 83% decrease (*p* < 0.05) in the selenium content in mycelium as compared with Series I (Figure 2b). In the presence of an excess of selenites, the selenium absorption was almost as effective as in Series I (decrease by about 10%, *p* < 0.05) (Figure 2b). Selenium absorption decreased when the medium contained a constant amount of selenite (0.2 mM) and the concentration of zinc rose from 0 to 0.8 mM (Series IV) (Figure 2c). Selenium absorption dropped from 311 to 45 (86%, *p* < 0.05). The greatest decrease in the concentration of selenium in the mycelium (by 74%) was observed when the zinc concentrations in the culture medium were 0.1–0.2 mM, thus providing selenite/zinc(II) molar ratios of 2:1 and 1:1, respectively. Further increases of the concentration of zinc up to 0.8 mM (affording a 4:1 ratio of selenite/zinc(II)) did not further reduce the selenium content (Figure 2c).

#### 3.1.3. Zn(II) Accumulation Exclusively versus in the Presence of SeO_3_^2−^

**Figure 3:**
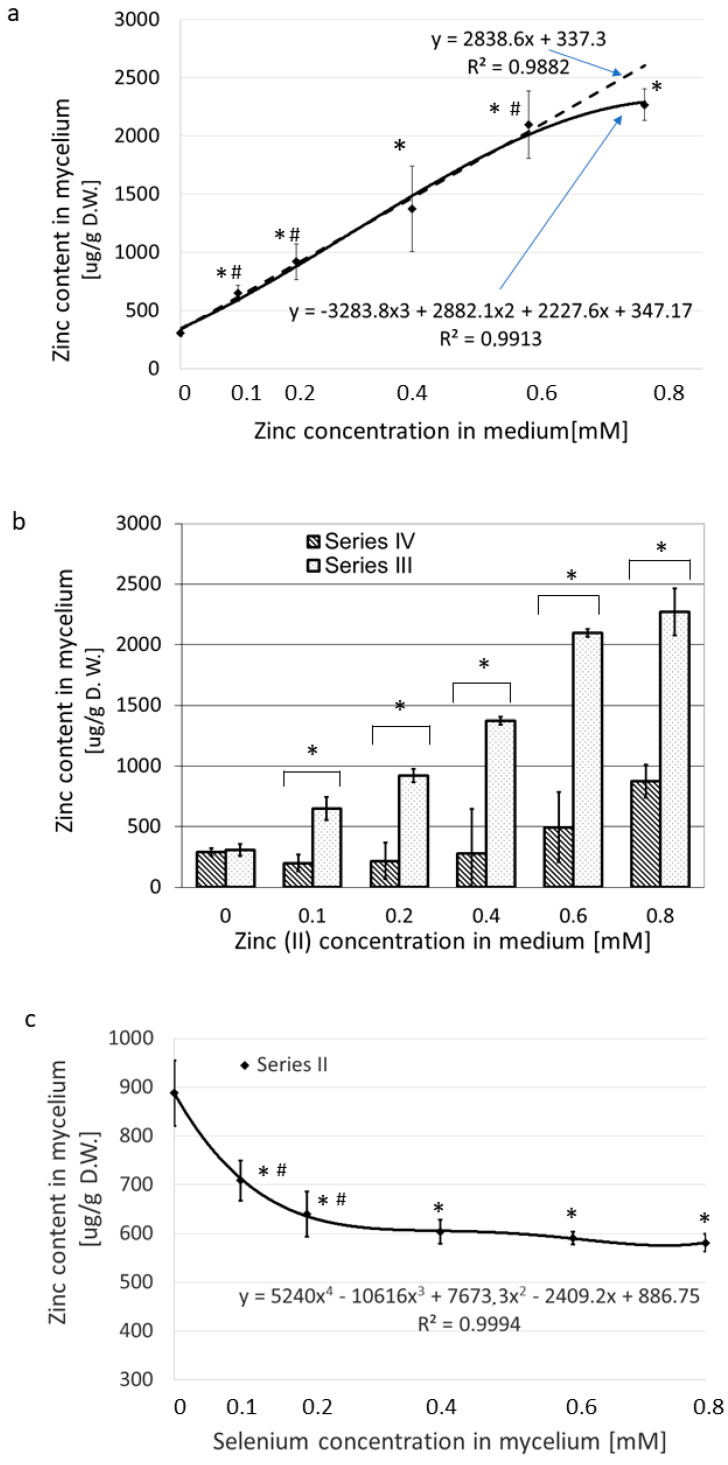
Accumulation of zinc in the mycelium of *L. edodes* from a medium enriched in Zn(II) exclusively versus in the presence of selenites (0–0.8 mM Se). (**a**) The effect of increasing the zinc content in the medium on the amount of Zn accumulated in the mycelium. The culture medium contained only zinc bromide and no added selenium. (*) values significantly different to the reference culture cultivated in non-enriched medium (*p* < 0.05). (#) values significantly different to the previous culture supplemented with a lower concentration of Zn(II) (*p* < 0.05). (**b**) Comparison of Zn accumulation into the mycelium when the medium was enriched exclusively with Zn(II) (Series III) versus when 0.2 mM sodium selenite was also present in the medium (Series IV). (*) values of Series III significantly different to Series IV (*p* < 0.05). (**c**) Impact of increasing selenite concentration in the medium on zinc accumulation from a medium enriched in 0.2 mM zinc bromide (Series II). (*) values significantly different to the reference culture cultivated in non-enriched medium (*p* < 0.05). (#) values significantly different to the previous culture supplemented with a lower concentration of Zn(II) (*p* < 0.05). The data are presented as the mean ± S.D. of five observations.

Figure 3a and Table S2 show the dynamics of zinc absorption when Zn alone was added to the medium in increasing concentrations (Series III). When the concentration of ZnBr_2_ in the medium increased from nearly zero to 0.8 mM, the values of absorbed zinc rose from 308 to 2370 µg/g of dry weight. The content of zinc in the mycelium correlated well with its concentration in medium in the concentration range 0.1–0.6 mM (Figure 3a).

The concentrations higher than 0.6 mM zinc bromide hampered Zn uptake from the medium. This effect was similar to that recorded for selenium uptake and suggests feedback inhibition at excessive concentrations, although another mechanism, such as saturation, may also be possible. When Na_2_SeO_3_ was held at a constant concentration of 0.2 mM in the medium and the ZnBr_2_ concentration was increased from nearly zero to 0.8 mM (Series IV), we noted an initial drop in zinc absorption into the mycelium, but the change was not significant (Figure 3b, Series IV.) Under these conditions, as the Zn content of the medium increased, the absorbed level of Zn also increased by nearly threefold in the medium supplemented with 0.8 mM ZnBr_2_.

When the medium contained a fixed amount of zinc(II) (0.2 mM) and the concentration of selenium varied from 0 to 0.8 mM (Series II), the values of absorbed zinc dropped from an initial high of 888 to as low as 591, approximately 25% (*p* < 0.05) (Figure 3c). Under these experimental conditions, the greatest decrease in the concentration of zinc in the mycelium was observed at the lower selenium levels, which included 0.1 and 0.2 mM of selenite in the culture medium, with zinc(II)/selenite molar ratios of 2:1 and 1:1. Further increases in the concentration of selenium of up to 0.8 mM (fourfold excess relative to the concentration of zinc(II)) did not reduce the absorbed zinc content significantly (Figure 3c).

### 3.2. Composition of the Water Extracts

Table 3 presents the data on the concentration of selenium, zinc, and saccharides in the *L. edodes* mycelial hot water extracts. The results are given in comparison with the Zn and Se concentration in the extracted mycelia.

### 3.3. Immunological Activity of Mycelial Extracts

Effective immune response to infection depends on the activation of immunocompetent cells [46]. Activated T lymphocytes express on their surface numerous molecules, including CD69 and CD25 (also known as interleukin-2Rα) [47]. CD69 is the earliest-acting antigen, upregulated four hours after activation. It is essential for T cell proliferation and survival. CD25 is upregulated 12–24 h after activation and initiates cell proliferation and differentiation [48,49,50].

**Figure 4:**
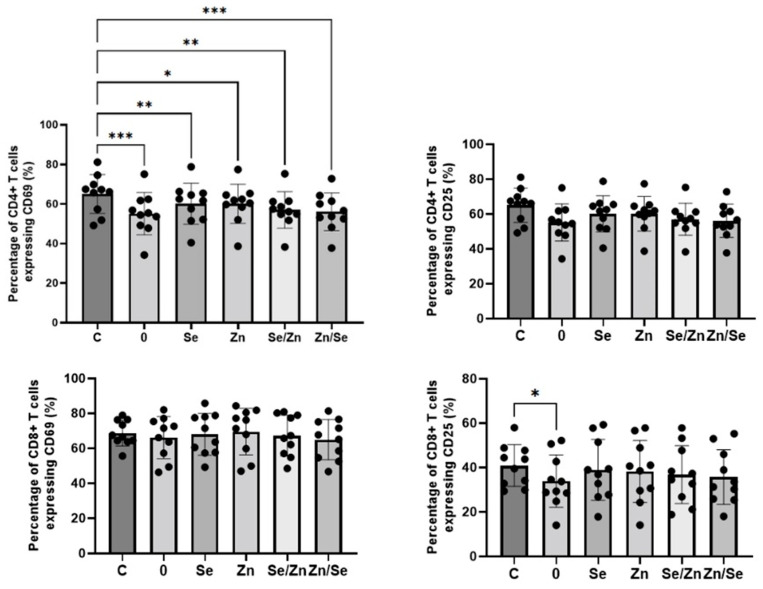
Effects of *L. edodes* mycelium water extracts with different concentrations of selenium and zinc on the activation of human CD4+ (**top row**) and CD8+ (**bottom row**) T cells stimulated with anti-CD3 Ab. PBMCs from seven donors were stimulated and treated with extracts at a concentration of 100 µg/mL for 24 h. The expression of CD69 and of CD25 markers on the surface of T cells were detected via flow cytometry. Statistical differences were considered when *p* < 0.05. * *p* < 0.05; ** *p* < 0.01; *** *p* < 0.001.

The effects of mycelial extracts on the expression of activation markers on CD4+ and CD8+ T cells stimulated with anti-CD3 Ab are presented in Figure 4. All analyzed extracts significantly downregulated the percentage of CD4+CD69+ T cells; however, extracts not enriched with micronutrients (Sample 0) showed the strongest effect in comparison to the control (C: 65.07 ± 9.83%; Sample 0: 55.11 ± 10.68%, *p* = 0.0004; Sample Se: 60.11 ± 10.39%, *p* = 0.0049; Sample Zn: 60.10 ± 9.90, *p* = 0.0358; Sample Se/Zn: 57.00 ± 9.19%, *p* = 0.0028; Sample Zn/Se: 56.07 ± 9.53%, *p* = 0.0008). A statistically significant impact on CD25 expression on CD4+ T cells was not observed (*p* > 0.05); however, this marker tended to be downregulated by the non-enriched extract. None of the analyzed extracts affected the expression of the CD69 marker on CD8+ T cells (*p* > 0.05), but the non-enriched fraction showed a significantly downregulated percentage of CD8+CD25+ T cells (40.84 ± 9.36% vs. 33.90 ± 11.78%, *p* = 0.0406).

**Figure 5:**
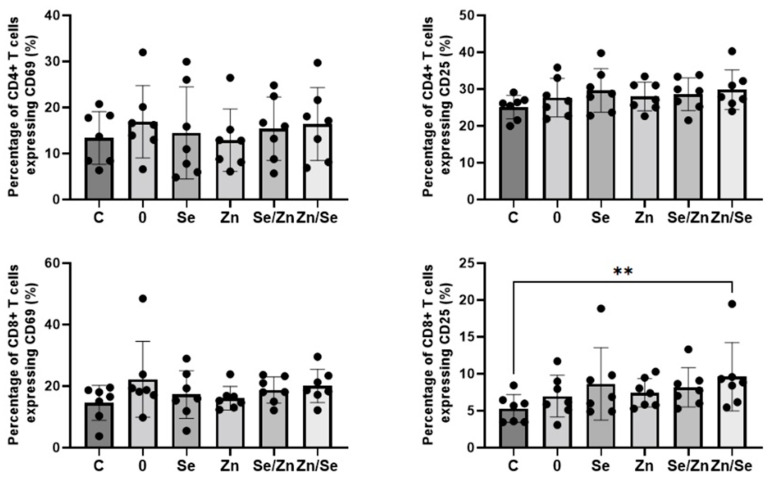
Effects of *L. edodes* mycelium water extracts with different concentrations of selenium and zinc on the activation of human CD4+ (**top row**) and CD8+ (**bottom row**) T cells stimulated with anti-CD3/CD28 Abs. PBMCs from seven donors were stimulated and treated with extracts at a concentration of 100 µg/mL for 24 h. The expression of the CD69 and CD25 markers on the surface of T cells was detected via flow cytometry. Statistical differences were considered when *p* < 0.05. ** *p* < 0.01.

The effects of *L. edodes* mycelial extracts on the expression of activation markers on CD4+ and CD8+ T cells stimulated with anti-CD3/CD28 Abs are presented in Figure 5. When cells were stimulated with a dual signal, the number of CD4+ T cells expressing the CD69 and CD25 markers was higher; however, the observed changes were not statistically significant (*p* > 0.05). None of the analyzed extracts were shown to have an effect on the expression of the CD69 marker on CD8+ T cells (*p* > 0.05), but the extract enriched with Zn and Se (sample Zn/Se) showed a significantly increased number of CD8+CD25+ T cells (5.28 ± 1.89% vs. 9.60 ± 4.63%, *p* = 0.0017).

## 4. Discussion

As stated in the introduction, the first objective of our study was to optimize the conditions for the simultaneous accumulation of zinc and selenium in the mycelial cultures of *L. edodes*, to obtain an immune-active preparation containing β-D-glucans and two micronutrients necessary for the proper functioning of the immune system. The mutual blockade of the transport of selenium and zinc to animal and plant cells is a well-known phenomenon, although its mechanism is not entirely clear [51]. Thus, we planned to check whether such a phenomenon, which could hinder the achievement of the assumed goal (obtaining a mycelium enriched in both organic selenium and zinc compounds), occurs in submerged cultured fungal cells.

As for the mutual influence of selenites and zinc(II) ions on the accumulation of Se and Zn in *L. edodes* mycelial cultures, the cumulation of selenium in the presence of zinc and of zinc in the presence of selenium strongly decreased in comparison to the amount of each ion absorbed when the medium was supplemented with that ion alone (Figure 2 and Figure 3). The mutual inhibitory effect of zinc ions and selenites on transport to the fungal cell is therefore similar to the effects described for other organisms [51]. It was confusing, however, that the strongest indication came from the results obtained at a Zn2+-to-SeO_3_^2−^ concentration ratio of 1:1 (0.2 mM:0.2 mM). Further, the substantial excess of the second ion did not cause a proportional inhibition of the accumulation process (Figure 2 and Figure 3). Since the molar ratio of ions in the culture medium influenced the accumulation of selenium and zinc in fungal cells more significantly than the absolute values of ion concentrations, we hypothesized that interactions between ions may be responsible for this phenomenon. We assumed that the formation of the zinc–selenite complexes of different charges would very likely inhibit the first stage of the accumulation process, i.e., biosorption. This hypothesis was confirmed in our further research; however, due to the extensiveness of the material, this will be described in a separate publication.

Our previous studies have shown that the optimum concentration of sodium selenite in the cultivation medium, providing that the *L. edodes* mycelium is enriched in organically bounded selenium, was 0.12–0.25 mM (10–20 µg of Se/L) [44,52]. To provide the same selenium content to the mycelium when zinc (II) ions are present in the medium, the culture needs to be enriched in a substantially higher amount of sodium selenite (above 0.4 mM). At this concentration of selenite, the strength of zinc ions should be lower than 0.4 mM to avoid zinc selenite precipitation (the ion product would not exceed the solubility product of zinc selenite). Taking into consideration the strong inhibitory effect of equimolar concentrations of selenites and zinc(II) on accumulation of both elements, a zinc concentration of 0.2–0.3 mM should be considered optimum. However, the amount of Zn in the mycelium cultured under these conditions will be much lower than in cultures supplemented with zinc salts exclusively.

Further experiments were aimed at examining whether the enrichment of the *L. edodes* preparations with selenium and zinc would cause a stronger immunomodulatory effect than in each of these elements separately. Thus, for the preparation of the hot water extracts subjected to the immunological experiments, mycelia enriched exclusively in selenium, exclusively in zinc, and in both elements at two different concentrations were selected (Table 3). The choice of the Zn(II) and SeO_3_^2−^ concentration ratio in the culture medium (0.8:0, 0.8:0.2, 0.2:0.8, 0:0.8 mM) resulted from the data presented above (Section 3.1.3).

The results of the ICP-MS analysis showed that the concentrations of Se and Zn in the extracts were almost linearly dependent on their concentrations in the mycelium (Table 3, Figure 5). However, they were almost 1000 times higher in the extracts than in the mycelium (mg/g versus µg/g). To sum up: the hot water extraction of the derivatives of selenium and zinc from the *L. edodes* mycelium was effective. Nevertheless, with high concentrations of Se and Zn in the mycelium, the extractability of these elements clearly decreased. This is probably a consequence of the ions binding to the structure of non-extractable proteins and polysaccharides.

The concentration of polysaccharides in the aqueous extracts clearly depended on the concentrations of zinc and selenium in the mycelium. The highest concentration was found in the extracts from the mycelium not supplemented with Se and/or Zn. Zinc supplementation had a much weaker effect on the concentration of polysaccharides in extracts than selenium supplementation. The lowest concentration of polysaccharides was found in extracts from mycelium supplemented with selenium alone. In our previous studies, we found that the supplementation of cultures with high concentrations of selenium changed the structure of the fungal cell wall, significantly reducing the content of extractable polysaccharides. The current results confirm these findings. The results obtained via immunological tests suggest that the highest biological activity occurs in *L. edodes* water extracts not supplemented with Se or Zn (Sample 0) or those where mycelium was enriched with a Se concentration of 0.2 mM and Zn at a concentration of 0.8 mM (Sample Zn/Se). Both analyzed T cell populations are essential with regard to protective immunity against viral infections, including SARS-CoV-2 [53,54] In our previous study, we demonstrated that the direction of *L. edodes* polysaccharides biological activity depends on the type of cell stimulation [43]. The present study confirms these observations. When cells were stimulated with anti-CD3 Ab only, extracts showed decreased T cell activation. In contrast, when cells were stimulated with a dual signal (anti-CD3/CD28 Abs), which is more physiological [55,56], the extracts showed enhanced activation. In conclusion, there is an insufficient amount of evidence to confirm whether Se or Zn supplementation to mycelial cultures of *L. edodes* enhances the immunomodulatory properties of water extracts. Therefore, future studies on larger samples focusing on the identification of intracellular signaling pathways are needed.

As mentioned in the introduction, the preparations obtained from *L. edodes*, mainly polysaccharides, are used in clinical practice to support the treatment of cancer and some other diseases resulting from reduced immunity (including infectious diseases). Several other types of products are derived from *L. edodes*; dried and pulverized fruiting bodies, hot water and alcohol extracts of fruiting bodies, biomass or extracts of mycelia, and the broth harvested from submerged liquid cultures are also widely used as functional foods or dietary supplements. They contain immune system enhancers but also other well-studied compounds that inhibit blood aggregation, reduce cholesterol levels, and exhibit antibacterial and antiviral effects. Enriching them with microelements that have a synergistic effect seems to be a sensible idea. The results of the research described in this paper indicate that although the idea of supplementing with zinc and/or selenium preparations obtained from *L. edodes* is not false, the problem requires further comprehensive research. Importantly, the ratio of polysaccharide/zinc/selenium concentration is crucial for both the mechanisms of immunomodulating action and the biological effect of these preparations.

## 5. Conclusions

We have successfully optimized the composition of the culture medium to obtain an *L. edodes* mycelia enriched in Se and Zn at the assumed concentrations. The molar ratio of ions in the culture medium influenced the accumulation of selenium and zinc in fungal cells more significantly than the absolute values of ion concentrations. The obtained results indicate that interactions between selenate and zinc ions in the culture medium (complexation) influence the process of accumulation. This has an impact on the final concentration of microelements in biomass and in the designed dietary supplements.

In the mycelial preparations (water extracts) tested in the present study, the content of selenium and zinc was proportional to the content in the mycelium, although a significant proportion of Zn and Se was present in the mycelium in a form that was not water-extractable(probably bound to proteins).

The immunological activity direction of the mycelial extracts strongly depends on the type of cell stimulation. More importantly, the effect of the mycelial extracts containing polysaccharides as well as zinc and seleniumon on the activation of T lymphocytes indicates the key role of fungal polysaccharides: the strongest effect was shown by preparations containing the highest concentration of these compounds, regardless of the presence of selenium and zinc. In the case of preparations enriched in selenium and zinc, a significant predominance of the concentration of zinc over the concentration of selenium seems to be more advantageous. The selenium and zinc contents of the examined preparations modified the immunomodulatory activity of mycelial polysaccharides; however, the mechanisms of action of various active ingredients of the mycelial extracts seem to be different. In our opinion, however, there is an insufficient amount of evidence to confirm whether Se or Zn supplementation to mycelial cultures of *L. edodes* significantly enhances the immunomodulatory properties of water extracts. Therefore, future studies on larger samples focusing on the identification of intracellular signaling pathways are needed. To sum up, the idea of enriching preparations isolated from the fungus *L. edodes*, widely used in drugs, dietary supplements, or functional foods, with zinc and selenium compounds acting synergistically with fungal polysaccharides is not wrong, but it requires further extended research.

## Figures and Tables

**Table 1:** The protocol of supplementation of culture media in Series I–IV.

Series	Precursor	Concentration of Precursor [mM]				
I	SeO_3_^2−^	0.1	0.2	0.4	0.6	0.8
	Zn^2+^	0	0	0	0	0
II	SeO_3_^2−^	0.1	0.2	0.4	0.6	0.8
	Zn^2+^	0.2	0.2	0.2	0.2	0.2
III	Zn^2+^	0.1	0.2	0.4	0.6	0.8
	SeO_3_^2−^	0	0	0	0	0
IV	Zn^2+^	0.1	0.2	0.4	0.6	0.8
	SeO_3_^2−^	0.2	0.2	0.2	0.2	0.2

**Table 2:** Instrumental conditions of ICP-MS.

Parameter	Value
RF Power	1550 W
Plasma argon flow rate	15 L min^−1^
Auxiliary argon flow rate	0.9 L min^−1^
Nebulizer argon flow rate	1.03 L min^−1^
Cell gas flow rate	4.3 mL min^−1^
Dwell time	0.3 s
Sweeps	100
Number of readings per replicate	3
Conditions	Oxide 156/140 and doubly charged 70/140 < 2%

**Table 3:** The composition of the extracts subjected to biological activity tests.

Sample Acronym	Molar Ratio of Medium Supplements		Concentration of Se and Zn in Mycelium		Concentration of Se, Zn, and Saccharides in Mycelial Water Extracts		
	(mM)		(ug/g)		(ug/g)	(ug/g)	(mg/g)
	SeO_3_^2−^	Zn (II)	Se	Zn	Se	Zn	Saccharides
0	0	0	2.52 (0.17)	298.10 (29.02)	0.39 (0.06)	92.65 (0.91)	196.40 (6.91)
Se	0.8	0	1553.91 (102.1)	181.92 (19.46)	203.60 (5.18)	134.81 (4.42)	139.64 (4.31)
Se/Zn	0.8	0.2	1395.89 (16.86)	591.25 (18.41)	199.39 (7.99)	152.04 (6.07)	146.60 (2.90)
Zn/Se	0.2	0.8	44.59 (6.72)	874.94 (194.16)	26.67 (0.56)	212.42 (4.42)	142.16 (9.72)
Zn	0	0.8	3.01 (0.65)	2370.32 (235.49)	7.07 (0.09)	393.15 (4.46)	188.53 (4.64)

Standard deviations are given in parentheses.

## Data Availability

The data presented in this study are available on request from the corresponding author and co-authors.

## Supplementary Materials

The following supporting information can be downloaded at: https://www.mdpi.com/article/10.3390/nu15184015/s1, Table S1: The total selenium content (µg/g) in mycelial dry mass in experiment series I-IV; Table S2: The total zinc content (µg/g) in mycelial dry mass in experiment series I-IV.
