# Supplementary material for: Optimization of Se- and Zn-Enriched Mycelium of Lentinula edodes (Berk.) Pegler as a Dietary Supplement with Immunostimulatory Activity
Source: Nutrients. 2023 Sep 16;15(18):4015. doi: 10.3390/nu15184015 (PMC10535943; doi:10.3390/nu15184015)
Supplement: Supplementary file 1 [file nutrients-15-04015-s001.zip › nutrients-2567913-supplementary.pdf]

SUPPORTING INFORMATION
The additional tables to which reference is made in the text are as follows:

Table S1. The total selenium content (µg/g) in mycelial dry mass in experiments series I-IV.

Series I			Series II			Series III			Series IV		
**Medium supplements [mM]**		**Selenium content [µg/g] (S.D.)**	**Medium supplements [mM]**		**Selenium content [µg/g] (S.D.)**	**Medium supplements [mM]**		**Selenium content [µg/g] (S.D.)**	**Medium supplements [mM]**		**Selenium content [µg/g] (S.D.)**
SeO_3^{2-}	Zn^{2+}		SeO_3^{2-}	Zn^{2+}		SeO_3^{2-}	Zn^{2+}		SeO_3^{2-}	Zn^{2+}	
0	0	2.52$_a$ (0.17)	0	0.2	0.75$_a$ (0.28)	0	0	1.2$_a$ (0.78)	0.2	0	310.84$_a$ (84.59)
0.1	0	73.09$_b$ (16.72)	0.1	0.2	57.34$_b$ (2.23)	0	0.1	3.43$_a$ (1.1)	0.2	0.1	139.82$_b$ (57.40)
0.2	0	380.31$_c$ (24.97)	0.2	0.2	73.52$_c$ (10.16)	0	0.2	1.83$_a$ (1.1)	0.2	0.2	80.83$_c$ (4.16)
0.4	0	904.31$_d$ (70.62)	0.4	0.2	344.10$_d$ (48.65)	0	0.4	1.22$_a$ (0.88)	0.2	0.4	58.39$_d$ (12.65)
0.6	0	1518.44$_e$ (96.42)	0.6	0.2	1256.31$_e$ (120.06)	0	0.6	1.90$_a$ (0.92)	0.2	0.6	52.74$_d$ (4.6)
0.8	0	1553.91$_e$ (102.17)	0.8	0.2	1395.89$_e$ (16.86)	0	0.8	3.01$_a$ (0.65)	0.2	0.8	44.59$_d$ (6.76)

Standard deviations (SD) are given in brackets, n=5 replicates Values in the same column bearing different letters were significantly different ($p< 0.05$).

Table S2. The total zinc content (µg/g) in mycelial dry mass in experiments series I-IV.

Series I			Series II			Series III			Series IV		
**Medium supplements [mM]**		**Zinc content [µg/g] (S.D.)**	**Medium supplements [mM]**		**Zinc content [µg/g] (S.D.)**	**Medium supplements [mM]**		**Zinc content [µg/g] (S.D.)**	**Medium supplements [mM]**		**Zinc content [µg/g] (S.D.)**
SeO_3^{2-}	Zn^{2+}		SeO_3^{2-}	Zn^{2+}		SeO_3^{2-}	Zn^{2+}		SeO_3^{2-}	Zn^{2+}	
0	0	298.10$_a$ (29.02)	0	0.2	887.82$_a$ (67.12)	0	0	308.31$_a$ (32.89)	0.2	0	288.96$_a$ (50.83)
0.1	0	210.64$_b$ (16.66)	0.1	0.2	708.57$_b$ (41.68)	0	0.1	648.89$_b$ (70.89)	0.2	0.1	200.15$_a$ (97.07)
0.2	0	288.96$_c$ (50.97)	0.2	0.2	639.57$_c$ (45.95)	0	0.2	920.28$_c$ (152.99)	0.2	0.2	217.05$_a$ (55.07)
0.4	0	195.71$_d$ (7.95)	0.4	0.2	603.41$_c$ (24.20)	0	0.4	1372.89$_c$ (367.10)	0.2	0.4	278.22$_a$ (33.31)
0.6	0	151.87$_d$ (42.12)	0.6	0.2	590.60$_c$ (13.17)	0	0.6	2097.30$_d$ (290.13)	0.2	0.6	494.77$_b$ (31.28)
0.8	0	181.92$_d$ (19.46)	0.8	0.2	591.25$_c$ (18.41)	0	0.8	2370.32$_d$ (235.44)	0.2	0.8	874.94$_c$ (194.16)

Standard deviations (SD) are given in brackets, n=5 replicates. Values in the same column bearing different letters were significantly different ($p< 0.05$).
